# Acute Effects of Muscle Flexibility and Myofascial Release of the Posterior Lower-Leg Muscles on Ankle Function in Individuals with Active Ankle Dorsiflexion Range of Motion Deficits

**DOI:** 10.3390/sports14040154

**Published:** 2026-04-15

**Authors:** Maria Giannioti, Konstantinos Fousekis, Eleftherios Paraskevopoulos, Dimitris Mandalidis

**Affiliations:** 1 Sports Physical Therapy Laboratory, Department of Physical Education and Sports Science, School of Physical Education and Sports Science, National and Kapodistrian University of Athens, 17237 Athens, Greece; gianniot@phed.uoa.gr (M.G.); elparaskev@phed.uoa.gr (E.P.); 2 Therapeutic Exercise and Sports Rehabilitation Laboratory, Physiotherapy Department, University of Patras, 26504 Patras, Greece; kfousekis@upatras.gr

**Keywords:** ankle dorsiflexion, self-stretching, foam rolling, IASTM, postural control, roll-off analysis, isometric strength, posterior lower-leg

## Abstract

Ankle dorsiflexion range of motion (ADF-ROM) deficits has been linked to impaired function, altered gait, and injury risk. This study’s objective was to examine the acute effects of static self-stretching (SSS), foam rolling (FR), and instrument-assisted soft tissue mobilization (IASTM) of the posterior lower-leg on ADF-ROM and functional ankle outcomes in individuals with ADF-ROM deficits. Thirteen healthy, physically active college students with active ADF-ROM ≤ 13°, assessed in a non-weight-bearing position, completed all three interventions in a randomized, within-subject repeated-measures design. Pre- and post-intervention assessments included ADF-ROM, ankle plantar flexor isometric strength (APF-IS), single-leg countermovement vertical jump (SLCVJ), anterior reach distance in the Y-Balance Test (A-YBT), and gait parameters (contact time and plantar pressure). A two-way repeated-measures ANOVA with Bonferroni post hoc tests was used. Effect sizes reported as partial eta squared ($\eta_{p}^{2}$) and Cohen $d_{z}$. All interventions significantly improved ADF-ROM (*p* < 0.001; $\eta_{p}^{2}$ = 0.885), with IASTM showing the largest increase (50.7%, $d_{z}\;$= 2.15), followed by FR (35.4%, $d_{z}$ = 2.20) and SSS (21.5%, $d_{z}$ = 1.82). Differences between IASTM and FR (*p* > 0.05, $d_{z}$ = 0.40) and between FR and SSS (*p* > 0.05, $d_{z}$ = 0.69) were nonsignificant, while IASTM was significantly greater than SSS (*p* < 0.05, $d_{z}$ = 0.92). Significant gains were also seen in A-YBT (*p* < 0.05; $\eta_{p}^{2}$ = 0.302) and rearfoot plantar pressure (*p* < 0.01; $\eta_{p}^{2}$ = 0.482), although pairwise comparisons were nonsignificant and demonstrated small-to-moderate effect sizes ($d_{z}$ = 0.35–0.52). No significant changes occurred in APF-IS, SLCVJ, or contact time and mid- and forefoot plantar pressures during roll-off. In conclusion, all interventions improved ADF-ROM, with IASTM and FR being comparably effective. However, only slight improvements in dynamic balance and certain gait parameters were noted, with no effect on strength or power.

## 1. Introduction

The ankle joint plays a critical role in facilitating interaction between the body and the supporting surface during both daily and athletic activities, contributing substantially to force absorption, transmission, and locomotor efficiency [1]. Despite its functional demands and structural complexity, mobility restrictions, particularly reduced ankle dorsiflexion range of motion (ADF-ROM), are frequently observed in both clinical and healthy populations, including athletes. Limited ADF-ROM has been associated with a wide range of musculoskeletal pathologies across the kinetic chain. At the distal level, restricted dorsiflexion has been linked to ankle sprains, with individuals demonstrating weight-bearing lunge test values ≤ 34° being up to five times more likely to sustain such injuries [2], as well as chronic ankle instability [3], Achilles tendinopathy [4], metatarsal stress fractures [5], and plantar fasciitis [6]. Proximally, reduced ADF-ROM has also been implicated in knee pathologies, including patellar tendinopathy [7], patellofemoral pain syndrome [8], and anterior cruciate ligament (ACL) injuries [9]. Notably, athletes with ADF-ROM ≤ 36.5° exhibit a substantially greater incidence of patellar tendinopathy over one year compared to those with greater dorsiflexion capacity (18.5–29.4% vs. 1.8–2.1%) [10]. Furthermore, restrictions in ankle mobility have been associated with posterior thigh strains [11], low back pain [12], and even upper extremity injuries, such as scapular and elbow dysfunctions [13], highlighting the systemic impact of ankle joint limitations. In athletes, repetitive loading combined with inadequate recovery may contribute to a gradual reduction in ADF-ROM [14,15].

From a biomechanical perspective, restricted ADF-ROM alters lower-limb kinematics by limiting forward tibial progression during weight-bearing tasks, thereby promoting compensatory movement strategies throughout the kinetic chain [16,17,18,19]. These adaptations often include increased knee valgus, altered hip mechanics, and changes in trunk positioning, which may elevate mechanical loading on the knee, hip, and spine. Such compensatory patterns have been identified as key contributors to injury risk, particularly in relation to ACL injury mechanisms [1]. In addition to injury susceptibility, reduced ADF-ROM negatively affects functional performance, being associated with deficits in lower limb strength [20], impaired dynamic balance [21], and reduced capacity for explosive movements such as sprinting and jumping [22,23]. These impairments likely reflect both mechanical constraints and alterations in neuromuscular control, including changes in muscle–tendon stiffness and proprioceptive function.

To address these limitations, various therapeutic interventions have been proposed to improve ADF-ROM, primarily targeting posterior lower leg soft tissues, including the gastrocnemius–soleus complex and associated myofascial structures. Static self-stretching (SSS) is one of the most prescribed methods for enhancing flexibility and increasing soft tissue extensibility [24,25]. More recently, myofascial release techniques such as foam rolling (FR) have gained popularity due to their proposed effects on tissue compliance, pain modulation, and joint mobility [26,27]. Additionally, instrument-assisted soft tissue mobilization (IASTM) is used in clinical practice to mechanically stimulate the myofascial system and facilitate mobility improvements through targeted tissue manipulation [28]. From a mechanistic perspective, these interventions may exert their effects through distinct but potentially overlapping pathways. Static stretching is thought to primarily influence passive tissue properties and stretch tolerance, while foam rolling and IASTM may additionally affect fascial adhesions, enhance local circulation, and modulate neural factors such as pain perception and muscle activation [29,30,31].

Despite the widespread use of these interventions, there is limited evidence directly comparing their effectiveness, particularly regarding their acute effects on ADF-ROM [32,33,34]. Moreover, while some studies suggest that such interventions may influence functional performance outcomes such as muscle strength and balance, their specific and comparative effects on these parameters, particularly in individuals with restricted ankle dorsiflexion, remain poorly investigated [35,36,37,38].

Therefore, the purpose of the present study was to investigate the acute effects of SSS and myofascial release interventions, including FR and IASTM, applied to the posterior lower-leg soft tissues, on ADF-ROM and selected functional performance outcomes related to ankle joint functions. It was hypothesized that these interventions would exert differential effects on ankle mobility, thereby providing insight into their relative effectiveness in improving dorsiflexion range of motion while preserving or enhancing performance parameters.

## 2. Materials and Methods

### 2.1. Sample

Sample size calculation for a within-subjects repeated-measures ANOVA using G*Power 3.1 indicated that 19 participants were required. This was based on a moderate effect size (f = 0.25), an alpha level of 0.05, a power of 0.80, a correlation of 0.5 among repeated measures, and conservative nonsphericity assumptions. However, due to the limited availability of participants who met the strict inclusion criteria within the predefined timeframe, a total of 13 participants (9 males and 4 females) were included in the study. The study group had a mean (±standard deviation) age of 25.0 (±3.2) years, body mass of 72.9 (±12.3) kg, height of 173.9 (±9.0) cm, and body mass index of 24.0 (±2.9) kg·m^−2^. Participants were recruited through direct invitations delivered during undergraduate and postgraduate student classes, as well as through in-class announcements within the Department of Physical Education and Sport Science of the local University. Participants were included in the study if they demonstrated active ADF-ROM values of ≤13°, measured in a non-weight-bearing position with the knee extended. This cut-off value was selected based on previous research indicating that ankle dorsiflexion during level walking typically reaches approximately 10–15° during the stance phase, permitting forward tibial progression over the foot, with ~13° frequently reported as a representative mean value for unrestricted, healthy adult gait [39,40]. Including individuals with less than 13° allowed us to study those with potentially impaired walking mechanics, providing insight into compensation, dysfunction, and rehabilitation needs. The exclusion criteria for participation in the study included painful musculoskeletal syndromes (e.g., low back pain or patellofemoral pain syndrome), skin infections, balance disorders, injuries sustained during the year before participation, and vascular conditions (e.g., thrombosis, acute phlebitis, or varicose veins).

All participants willingly participated in the study and provided written informed consent after receiving both verbal and written explanations of the research objectives. The study protocol was reviewed and approved by the Research Bioethics Committee of the Department of Physical Education and Sport Science of the local university (Reg. No. 1221/16 September 2020).

### 2.2. Testing Procedure

A within-subject, repeated-measures design was employed to investigate the acute effects of SSS, FR, and IASTM targeting the posterior lower-leg on: (i) ADF-ROM, (ii) ankle plantar flexor isometric strength (APF-IS), (iii) explosive power by means of the single-leg countermovement vertical jump (SLCVJ), (iv) dynamic balance based on the anterior excursion of the Y-Balance Test (A-YBT), and (v) roll-off phase gait parameters. Dorsiflexion was assessed bilaterally at baseline, and the limb with the most restricted range of motion was selected for analysis. In the present sample, the most restricted dorsiflexion was observed in the dominant limb, which was identified using the Waterloo Footedness Questionnaire—Revised (WFQ-R) [41], a tool with established validity and reliability in the local population [42]. Each participant completed all three interventions in a randomized and counterbalanced order, with pre- and post-intervention assessments conducted at each experimental time point. A minimum interval of seven days between interventions was selected as a washout period to reduce potential residual effects from the preceding intervention, thereby minimizing carryover bias and allowing each condition to be evaluated under comparable baseline conditions [24,28,43,44]. Participants were instructed to continue their regular physical activity routines between sessions. As all participants were physical education students with relatively stable activity patterns, this approach was deemed appropriate to minimize variability and reduce potential confounding effects on the acute responses across experimental sessions.

### 2.3. Outcome Measures

**Figure 1:**
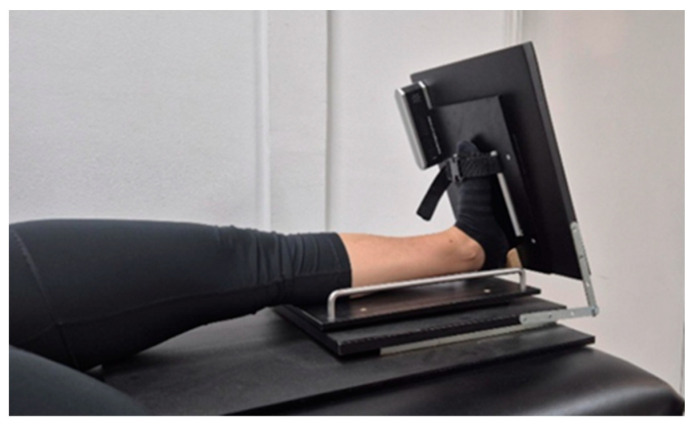
Assessment of active ankle dorsiflexion range of motion using a custom-made device.

Active ankle dorsiflexion range of motion was measured with the participant in a long sitting position on a treatment table, and the knee fully extended, using a custom-made device specifically designed to maintain the subtalar joint in its neutral position, thereby minimizing any potential influence during active open kinetic chain ankle movement [45] (Figure 1). Maximal ADF-ROM, defined as the greatest active, pain-free dorsiflexion movement that could be achieved by each participant, was measured using a digital inclinometer (Saunders Digital Inclinometer, The Saunders Group Inc., Chaska, MN, USA), an instrument previously validated and demonstrated to be reliable for assessing ADF-ROM [46]. Three trials were recorded, and the average value was used for data analysis.

**Figure 2:**
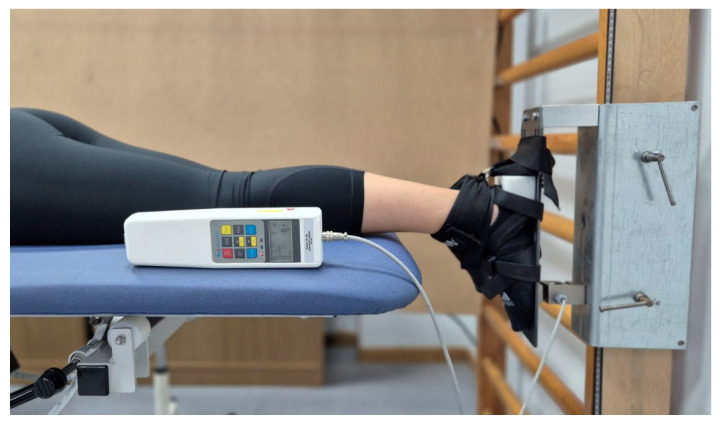
Subject and foot positioning for assessing maximal isometric strength of the ankle plantar flexors.

The APF-IS was measured using an S-type load cell dynamometer (dimensions: 76.2 × 51 × 28.2 mm; FH 5K, Sauter GmbH, Balingen, BLACKROLL AG, Bottighofen, Switzerland) connected to a data acquisition device. The acquisition device was interfaced with a computer for data recording, storage, and analysis using dedicated software. This model has a measurement range of 5 kN and a resolution of 1.0 N. For the present study, the dynamometer was stabilized on a custom-made metal frame mounted on a vertically oriented wooden element (2.0 m × 0.10 m), which was fixed to a wall bar (Swedish ladder) (Figure 2).

Strength testing was performed with the participant lying prone on a treatment table. The tested foot was positioned in a neutral (0°) plantarflexion/dorsiflexion position. The table height was adjusted to align the dynamometer with the heads of the metatarsal bones. Non-elastic straps were used to stabilize the foot on the frame. Each participant performed two submaximal familiarization contractions (at 50% and 75% of their maximal voluntary contractions), followed by three maximal voluntary isometric contractions lasting 5 s each. The APF-IS was calculated as the average of the peak values obtained during the three maximal attempts. A rest period of 1 min was allowed between attempts to ensure recovery. The reliability of APF-IS, as reported in a previous study, was excellent (ICC_3,2_ = 0.98) and considered suitable for clinical use [47].

Single-leg countermovement vertical jump was assessed using a 35 g inertial sensor with dimensions of 50 × 70 × 30 mm (Gyko, Microgate, Bolzano, Italy). This sensor combines a gyroscope, a triaxial accelerometer, and a magnetometer, and it is designed to analyze the motion of individual body segments. Data were acquired at a sampling frequency of 1000 Hz and processed using the manufacturer’s built-in sensor-fusion algorithm, integrating accelerometer and gyroscope data to estimate three-dimensional orientation. The magnetometer was enabled under default settings, and data were collected in an environment free of substantial magnetic interference. No additional external filtering was applied beyond the device’s internal processing framework. The reliability and validity of vertical jump measurements have been previously established and are considered suitable for clinical application [48,49].

Prior to data collection, the inertial measurement unit was calibrated using a standardized static and alignment procedure. The device was placed on a stable horizontal surface to correct accelerometer and gyroscope offsets, followed by alignment to the anatomical reference frame. Signal integrity was verified via real-time inspection before each measurement session, after which data were transmitted wirelessly to a computer via Bluetooth and subsequently stored, visualized, and analyzed using the Gyko Repower software (version 1.1.1.10, Microgate, Bolzano, Italy). The sensor was affixed to the participant’s lower back (lumbar region) using a specialized belt.

Each participant performed a SLCVJ on the tested limb while wearing their own athletic footwear and keeping their hands on the iliac crests to minimize arm swing. Before testing, three submaximal practice trials were performed to familiarize the participants with the procedure and ensure consistency in effort and technique. Subsequently, three maximal attempts were recorded, with a 30-s rest interval between trials.

**Figure 3:**
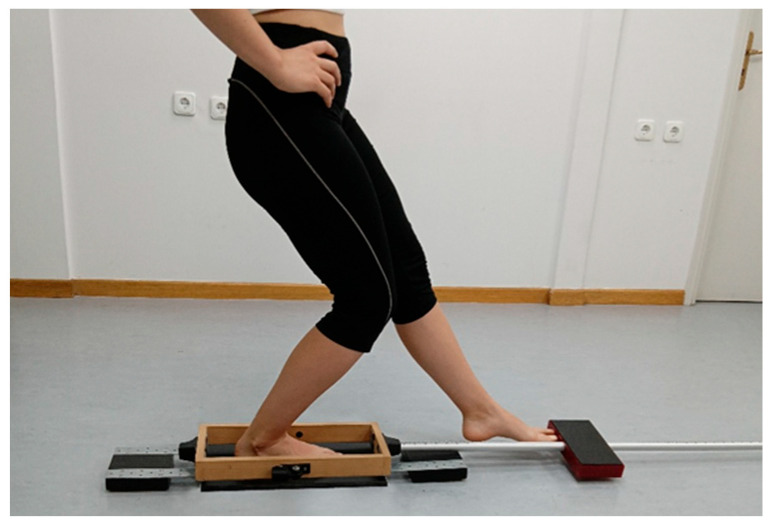
Final position during dynamic balance assessment.

Dynamic body balance was assessed using a 10-s anterior reach of the Y-Balance Test (A-YBT), with participants standing barefoot and the tested limb placed on the floor. The A-YBT was performed using a custom-made device comprising a wooden frame, an aluminum tube mounted at the anterior edge, and a rectangular wooden block covered with acrylic plastic that slid along the tube, designed to closely resemble the standard Y-Balance Test apparatus The validity of the device has been established in previous studies, and the reliability of the A-YBT task has been shown to be acceptable for clinical use [50,51]. Each participant was instructed to reach forward as far as possible by sliding the wooden block along the tube with the non-tested limb, then return to their starting position without losing balance. At the same time, they were asked to keep their hands placed on the iliac crests and to keep the plantar aspect of their foot in contact with the platform (Figure 3).

The trial was repeated if the participant removed their hands from the iliac crests, lifted or moved their foot from the platform, or failed to return the reaching limb to its initial position at the end of the attempt. Dynamic body balance was assessed by measuring the maximum distance the participant could slide the block while reaching forward with the non-tested limb. This distance was then expressed as a percentage of lower limb length, defined as the distance from the anterior superior iliac spine to the medial malleolus, measured using a standard measuring tape. Each participant completed three familiarization trials followed by three recorded attempts, with the mean value of the attempts used for analysis.

Gait analysis was conducted by assessing foot roll-off using a pressure distribution platform (FDM-S Measuring System for Force Distribution, Zebris Medical GmbH, Isny im Allgäu, Germany). The platform measured 710 × 400 × 15 mm, contained 2560 pressure sensors, and operated at a sampling frequency of 120 Hz. It was connected via a wired interface to a computer, allowing for data recording, storage, and analysis through dedicated software (Win FDMS v.0.1 for Windows, Zebris Medical GmbH, Isny im Allgäu, Germany). Foot roll-off was defined as the gait phase during which the foot progresses from heel contact through mid-stance to toe-off, facilitating forward propulsion. Each participant was required to step with the lower limb under evaluation onto the platform, which was placed on the floor, while walking at a natural pace. To ensure valid data collection, the participants were allowed practice trials to estimate the required step length and accurately position their foot on the target area. A total of five valid trials were recorded.

### 2.4. Interventions

Each participant was asked to perform SSS exercises in a standing position on an adjustable inclined surface. The inclination angle was set to be 3° less than the participant’s maximal active ADF-ROM in the tested lower limb as measured in a standing lunge position [52]. After the stretching technique and correct execution were explained, the participants were instructed to stretch the muscles in the posterior compartment of the leg to the point of mild discomfort but not pain to avoid compromising muscle performance [53].

**Figure 4:**
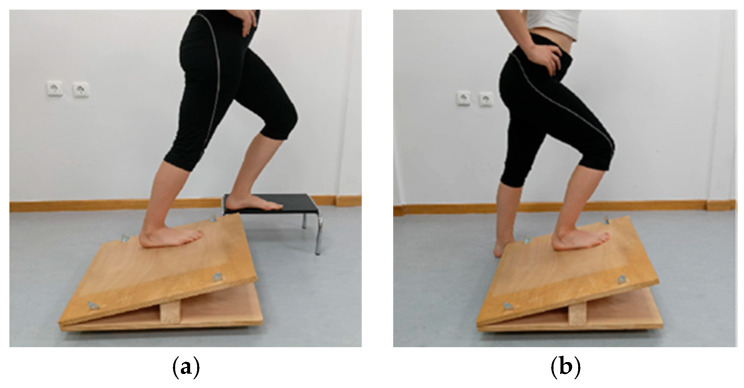
(**a**) Static self-stretch with the foot placed on an inclined surface and the knee extended. (**b**) Static self-stretch with the foot placed on an inclined surface and the knee flexed.

The stretching exercises were performed on the limb under evaluation, with the knee tested in both partial flexion and full extension. The contralateral limb was lightly supported either on the ground or on a step adjacent to the apparatus, while the participant used their hands to support themselves on a stable surface (Figure 4). The total duration of the stretching protocol was 6 min, consisting of three sets of 30 s for static stretching performed with the knee both flexed and extended, with 30-s rest intervals between sets. A 1-min rest was ensured before they switched from the knee-flexed stretch to the knee-extended stretch [53].

**Figure 5:**
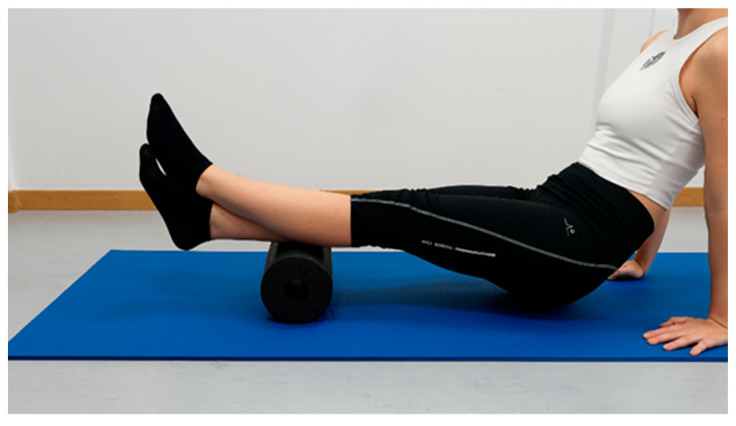
Foam rolling on the medial posterior surface of the lower leg.

Foam rolling was performed using the Blackroll Standard Foam Roll (Blackroll, Germany), which measures 30 cm × 15 cm and weighs 152 g. The procedure was conducted on an exercise mat placed on the floor. The first author (M.G.), a certified therapist who specialized in FR, instructed the participants on the correct technique and the proper execution of the intervention. Initially, the participants familiarized themselves with the technique. Subsequently, they were asked to perform rolling on the posterior calf muscles in accordance with the therapist’s specific instructions. During the procedure, the leg undergoing rolling rested on the foam roller, while the contralateral leg was placed on top of the roller to increase the applied pressure. The participant used their arms to lift their body weight and performed linear movements over the roller (Figure 5). The rolling was repeated with the hip internally and externally rotated to target the medial and lateral heads of the gastrocnemius, respectively. The order of hip rotation (internal or external first) was randomized.

The intervention lasted 6 min, consisting of 3 sets of 30 s with the hip internally rotated to target the medial head of the gastrocnemius, and 3 sets of 30 s with the hip externally rotated to target the lateral head of the gastrocnemius. The rolling frequency was set at 15 beats per 30 s and paced using a metronome (2 s per beat), with 30-s rest intervals between repetitions and a 1-min rest before switching from internal to external rotation. Although the duration of a foam rolling (FR) intervention does not appear to significantly influence joint range of motion [54], the present program was designed in accordance with evidence indicating that rolling for up to 300 s per muscle and 450 s per session may enhance performance and range of motion [55].

**Figure 6:**
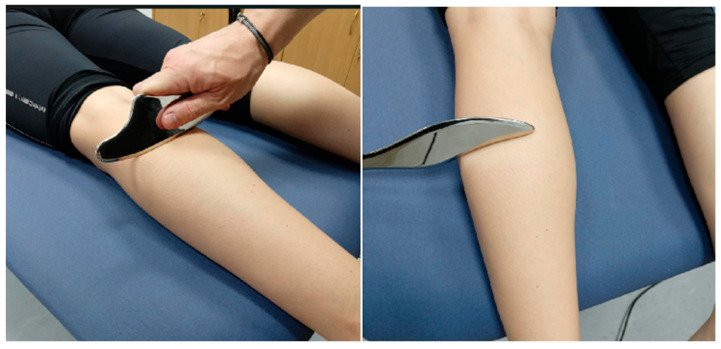
Application of the Ergon IASTM technique to the posterior compartment of the lower leg.

Soft tissue mobilization was performed by the first author (M.G.), a certified therapist trained in the Ergon IASTM technique, an instrumented technique universally used in soft tissue mobilization (https://ergontechnique.com/, accessed on 5 July 2025). The participant was first positioned in a comfortable and supported posture to allow full access to the targeted muscle group. The intervention was then performed using specific tools recommended by the Ergon IASTM procedure for mobilizing the soft tissue of the posterior compartment of the leg. Before application, a specially formulated emollient cream was applied to the treatment area to facilitate smooth gliding of the IASTM instruments over the skin. The first minute was dedicated to preparing the treatment area using an introductory technique (RUB) with a specialized tool (RHINO), aiming to warm up and stimulate the superficial fascia. This was then followed by the larger surface application technique (WAVE maneuver), which was used to linearly mobilize the soft tissues to create tissue glide and identify fascial restrictions. Subsequently, broader fascial mobilization was performed using a series of specific maneuvers applied in multiple directions with a specialized IASTM tool (the FASCIALIZER; Figure 6). These included the SNAKE technique, which consisted of repeated diagonal strokes intended to release myofascial adhesions along oblique lines of force; the RAZOR technique, which involved semicircular manipulations targeting localized areas of myofascial stiffness; and the GLOBE technique, which used circular mobilization patterns to release myofascial restrictions. Additionally, targeted techniques were employed to gently release the interface between the soleus fascia and the medial aspect of the tibia and its surrounding tissues, as well as to reduce localized adhesions in the Achilles tendon region. Tool movements were applied in multiple directions (parallel, transverse, and diagonal to the fiber orientation). The depth and pressure of the maneuvers were adjusted according to the participant’s tolerance. The application angles for RUB, WAVE, RAZOR, and GLOBE ranged from 30° to 60°. For consistency with the other interventions and in accordance with previous studies, the entire procedure lasted approximately six minutes [56].

### 2.5. Statistical Analysis

A two-way repeated measures ANOVA was conducted for each dependent variable, with time (pre- vs. post-intervention) and intervention (SSS vs. FR vs. IASTM) as within-subject factors. When statistically significant effects, either main or interaction, were observed, Bonferroni-adjusted pairwise comparisons were conducted to identify specific differences between conditions. However, when both main effects and interactions were significant, interpretation was restricted to the interaction effects.

Effect sizes for the main effects and interactions were quantified using partial eta squared ($\eta_{p}^{2}$) values classified as small ($\eta_{p}^{2}$ = 0.01), medium ($\eta_{p}^{2}\;$ = 0.06), or large ($\eta_{p}^{2}$ = 0.14) [57]. Effect sizes for pairwise comparisons were calculated as Cohen’s d for dependent samples ($d_{z}$), derived from the t-statistics of the paired comparisons $d_{z}=\frac{t}{\sqrt{n}}$ [58,59], with t-values obtained from the ratio of the mean difference to its standard error, based on the repeated-measures ANOVA output. Effect sizes were interpreted as trivial (<0.2), small (0.2–0.49), moderate (0.5–0.79), large (0.8–1.19), very large (1.2–1.99), and extremely large (≥2.0).

Before the analysis, all variables were assessed for normality using the Shapiro–Wilk test and for sphericity using Mauchly’s test of sphericity. When the assumption of sphericity was violated, the Greenhouse–Geisser correction was applied. All statistical analyses were performed using IBM SPSS Statistics (version 30, IBM Corp., Armonk, NY, USA). The level of significance was set at *p* < 0.05.

## 3. Results

Assessment of data normality conducted before the analyses indicated that the assumption of normality was not violated. The assumption of sphericity was violated only for the treatment effect on contact time, whereas violations of the time × treatment interaction were observed for contact time, midfoot plantar pressure, and A-YBT. The means and percentage change, calculated as condition—baseline/baseline × 100, and confidence intervals for all dependent variables measured in the study are presented in Table 1.

Statistical analysis revealed significant main effects of time [F (1,12) = 92.54, *p* < 0.001, $\eta_{p}^{2}$ = 0.885] and a significant interaction between time (pre- and post-intervention) and type of intervention (SSS vs. FR vs. IASTM) [F (2,24) = 11.723, *p* < 0.001, $\eta_{p}^{2}$ = 0.494] for ADF-ROM. Post hoc pairwise comparisons with a Bonferroni adjustment were conducted to control for Type I error, demonstrating significant post-intervention improvements in ADF-ROM across all muscle techniques targeting relaxations of the posterior lower-leg muscles (*p* < 0.001) with large effect sizes (Cohen $d_{z}$) observed for SSS ($d_{z}$ = 1.82), FR ($d_{z}$ = 2.20), and IASTM ($d_{z}$ = 2.15). IASTM demonstrated the greatest improvement (50.7%), without a significant difference compared to FR (35.4%, *p* > 0.05, $d_{z}$ = 0.40), but with a significantly greater improvement than SSS (21.5%, *p* < 0.05, $d_{z}$ = 0.92; see Table 1). A moderate effect was also observed between FR and SSS ($d_{z}$ = 0.69), although this comparison was not statistically significant (*p* > 0.05).

Significant main effects of time were revealed for normalized distance recorded during A-YBT [F (1,12) = 5.182, *p* < 0.05, $\eta_{p}^{2}$ = 0.302], and RF-PP exerted during roll off [F (1,12) = 11.172, *p* = 0.01, $\eta_{p}^{2}$ = 0.482], with values increasing following all interventions. Pre-to-post-intervention pairwise comparisons for the A-YBT revealed small-to-moderate effect sizes for SSS ($d_{z}$ = 0.35), FR ($d_{z}$ = 0.52), and IASTM ($d_{z}$ = 0.39); however, none of these changes reached statistical significance (*p* > 0.05). Similarly, pairwise comparisons between pre- and post-intervention assessments for RF-PP demonstrated small-to-moderate effect sizes across all interventions (SSS: $d_{z}$ = 0.50, FR: $d_{z}$ = 0.44, IASTM: $d_{z}$ = 0.49), although none of the observed changes were statistically significant (*p* > 0.05). Furthermore, neither the treatment main effects nor the time × treatment interaction was statistically significant.

No significant main effects of time or treatment, nor any significant time × treatment interactions, were observed for APF-IS, SLCVJ, or MD-PP and FF-PP.

## 4. Discussion

Although commonly used interventions targeting the posterior lower leg have been shown to improve ankle joint mobility, evidence regarding their effects on concurrent functional outcomes remains limited. This is particularly important in individuals with ADF-ROM restrictions, as improvements in ROM do not necessarily translate into enhanced performance. Therefore, the aim of this study was to investigate the acute effects of three commonly used interventions (SSS, FR, and IASTM), applied to the posterior lower leg, on ADF-ROM and ankle-related performance outcomes in individuals with restricted ADF-ROM (≤13°).

### 4.1. Ankle Dorsiflexion Range of Motion

A significant increase in active ADF-ROM was observed following all three interventions, with IASTM producing the most substantial gains (50.7%), followed by FR (35.4%) and SSS (21.5%) compared to baseline measurements. However, only the changes induced by IASTM were statistically significant compared to SSS. This finding is consistent with evidence summarized in previous systematic reviews examining the short-term effectiveness of SSS [25], FR [44], and IASTM [60,61] in improving ankle joint mobility. It aligns with earlier studies reporting comparable ADF-ROM improvements between SSS and FR [32,33], but contrasts with findings indicating FR may be superior to IASTM [34]. Moreover, the improvements observed in the present study were substantially greater than those typically reported in the literature. For instance, maximal increases in ADF-ROM of up to 17% (4.6°) have been reported following SSS performed to the end range of available passive motion (~25° dorsiflexion) [62]. Foam rolling has resulted in up to a 22% increase in passive dorsiflexion (6.0°) [63], while a 5-min IASTM intervention produced a 10.7% ± 10.8% improvement (3.4°) [60].

The relatively large percentage increases observed in ADF-ROM in our study are likely attributable to the use of active joint range measurements following stretching and myofascial release techniques, as opposed to the passive ROM assessments commonly employed in previous research [60,62,63]. Acute increases in passive ROM following a single session of static stretching have been largely attributed to enhanced stretch tolerance, reflecting the central nervous system’s increased ability to modulate discomfort during muscle elongation, as well as reduced musculotendinous stiffness [64]. Furthermore, stretching and myofascial release may influence fascial hydration and decrease connective tissue viscosity through thixotropic mechanisms, thereby reducing resistance to movement [65]. These effects are complemented by friction-induced increases in local tissue temperature, improved vascular perfusion, and stimulation of myofascial proprioceptors [65]. Additionally, static stretching has been shown to reduce muscle spindle excitability, thereby decreasing reflexive resistance to elongation during active movement [66]. Sustained pressure from myofascial techniques may also activate Golgi tendon organs, promoting autogenic inhibition and facilitating muscle relaxation [67]. Moreover, the contraction of antagonist muscles during active movement may induce reciprocal inhibition of the target muscle, further enhancing its capacity to lengthen [68]. These mechanisms may exert a more significant influence on active rather than passive range of motion, where neuromuscular responses are presumably less pronounced, and the capacity for muscle length change is inherently limited due to the already elongated muscle lengths at which these measurements are conducted. Collectively, these mechanical, neural, and proprioceptive effects support the hypothesis that interventions such as stretching and myofascial release may lead to greater improvements in active ROM compared to passive ROM.

The greater changes obtained with IASTM may be attributed to the mechanical properties of the technique, which allow for deeper and more localized fascial mobilization. These findings align with previous research suggesting that tool-assisted techniques can produce more immediate and pronounced changes in tissue extensibility and neuromuscular responses [28]. Additionally, the friction and shear forces generated by IASTM may enhance local circulation and stimulate mechanoreceptors, promoting both tissue pliability and central nervous system modulation [69]. These mechanical stimuli can increase blood flow, reduce tissue viscosity, and temporarily alter neuromuscular tone, facilitating improved tissue extensibility and joint motion [28]. Furthermore, stimulation of cutaneous and fascial mechanoreceptors may have produced neuromodulatory effects, enhancing proprioceptive feedback and contributing to the observed improvements in ROM [27,28]. Moreover, the superior ADF-ROM outcomes observed with IASTM may, in part, be attributed to its influence on latent myofascial trigger points (MTrPs) located in the gastrocnemius and soleus muscles, areas where MTrPs are among the most frequently reported in the lower limb [70]. This is supported by previous findings from Grieve et al. [71] who reported a 3.3° increase in ankle dorsiflexion ROM following a single session of trigger point pressure release targeting latent soleus MTrPs.

### 4.2. Muscle Strength and Power

Plantar flexor isometric strength remained unaffected by any of the interventions. Post-intervention mean strength values showed a slight, non-significant increase for SSS (%Δ = +2.6%), and slight decreases for both FR (%Δ = −2.3%) and IASTM (%Δ = −5.7%). Our findings are consistent with several review articles concluding that short-duration static stretching (≤60 s per muscle group), as employed in the present study (30 s), has only a trivial effect (%Δ = 1–2%) on subsequent strength and power performance, regardless of statistical significance [24,31,72]. In addition to dose-related effects, the strength changes observed in the present study may also have been influenced by the participant’s lower extremity position during strength assessment. Performing the measurements with the ankle in the neutral position and the knee extended may have elongated the gastrocnemius to a degree that limited the potential for detecting measurable adaptations, despite the use of isometric testing, a contraction type generally considered more sensitive to identifying strength changes following SSS [24]. This is likely because potential post-SSS strength changes are typically less pronounced when muscles are tested at longer lengths [24,73]. Several review articles have also concluded that foam rolling (FR) exerts negligible effects on muscle strength (%Δ = 1.8%) [74], with comparable findings reported for plantar flexor strength following a 4.5-min IASTM intervention targeting the calf musculature [38].

Furthermore, none of the interventions resulted in significant changes in explosive power, as measured by the SLCVJ. This finding aligns with the conclusions of most systematic reviews, which report that acute improvements in flexibility or mobility following SSS or myofascial release techniques such as FR do not necessarily translate into enhanced explosive performance outcomes, such as jumping ability [24,74,75]. On the other hand, findings regarding vertical jump performance following a single session of IASTM are conflicting. Some studies report no significant changes [76], which aligns with our results, while others have found significant improvements [38,77]. The conflicting findings in the literature may be explained by variations in how the IASTM technique was applied. Given that vertical jump performance relies on the coordinated activation of multiple muscle groups, including the posterior calf muscles, targeting only these muscles, as in the present study, may have limited the ability to affect other key muscle groups involved, which may explain the non-significant changes observed.

Alterations in muscle mechanical properties following short-term relaxation interventions are unlikely to meaningfully affect plantar flexor strength or power, as the associated mechanical changes and transient neural responses are too small to impair force transmission or motor drive. Acute static stretching has been shown to decrease passive resistive torque, muscle stiffness, and tendon stiffness, thereby reducing overall muscle–tendon unit (MTU) stiffness [64]. Increased compliance may shift the muscle’s operating range toward shorter sarcomere lengths on the force–length curve and may reduce the efficiency of force transmission [78]. Furthermore, reductions in passive stiffness have been associated with increases in electromechanical delay (EMD), suggesting that greater time is required to stretch the series elastic components before external force production occurs [79]. Computational modeling evidence further supports the mechanical relevance of tendon compliance, demonstrating that changes in tendon stiffness influence muscle force distribution and transmission characteristics [80]. Importantly, however, longitudinal and acute experimental data indicate that despite measurable reductions in musculotendinous stiffness, maximal force production is often preserved [81], reinforcing the concept that short-term mechanical alterations alone are insufficient to meaningfully impair strength performance.

In parallel, the absence of significant strength loss may be partly attributed to transient neural responses elicited by short-duration interventions. For example, brief static stretching has been shown to reduce spinal excitability, as demonstrated by Budini et al., who reported a temporary inhibition of the soleus H-reflex during passive dorsiflexion, with partial recovery at approximately 6 and 21 s, and full restoration immediately after returning to plantar flexion [66,82]. While this short-term inhibition may momentarily reduce force output during the stretch, it has negligible influence on strength performance afterward. Meta-analytical data further support that when static stretch repetitions are ≤30 s, decreases in maximal voluntary contraction (MVC) are trivial or non-significant [24,72]. Thus, despite momentary fluctuations in neural excitability, muscle strength remains functionally preserved.

Finally, despite FR and IASTM appearing to enhance ADF-ROM, primarily through neurophysiological and mechanotransductive mechanisms, these responses do not seem to translate into meaningful improvements in neuromuscular performance, as strength and power outcomes remain largely unchanged [60,74,83]. This apparent dissociation between gains in mobility and the absence of improvements in strength and power performance has been attributed to several mechanical and neuromuscular mechanisms. These mechanisms suggest that the magnitude and duration of the induced mechanical and neural alterations may be insufficient to modify force-generating capacity or motor unit recruitment patterns [24,27,74]. However, the current body of evidence remains inconclusive. As a comprehensive analysis of these mechanisms is beyond the scope of the present study, readers are referred to existing systematic and narrative reviews for a more detailed examination.

### 4.3. Dynamic Balance

The A-YBT results demonstrated a small but statistically significant main effect of time, indicating modest improvements in dynamic balance following all interventions with no technique demonstrating statistical superiority over the others. Furthermore, the observed small-to-moderate effect sizes suggest that, although performance improved, the magnitude of change was modest and may not translate into substantial functional or clinical benefits.

Similar findings have been reported following isolated SSS exercises targeting the plantar flexors, with improvements observed across all directions of the YBT, including the anterior reach [37]. Comparable results have also been noted following IASTM interventions [38]. In contrast, FR of the posterior calf muscles has shown no significant effects on balance in existing studies; however, these findings are limited to static assessments, such as the Stork test using a massage roller [84].

The Y-Balance Test, particularly the anterior reach used in the present study to assess dynamic balance, is known to be significantly influenced by ankle dorsiflexion range of motion [85,86], an ankle-specific capacity that was improved as a result of our intervention. Both Jung et al. [37], following SSS exercises, and Yana et al. [38], after IASTM on the plantar flexors, reported similar concurrent improvements in ADF-ROM and A-YBT performance. Other studies, however, have reported increased center of pressure (COP) area with or without changes in EMG in the stretch limb between pre-stretching and immediately unilateral post-stretching of the plantar flexor muscles during unipodal quiet standing [87,88]. These findings suggest that the improvements in A-YBT performance may have occurred once the mechanical limitation of ADF-ROM was reduced, despite reduced postural stability, as indicated in other studies by increased COP displacement and the need for enhanced neuromuscular control. Furthermore, dynamic balance improvements may be attributed to the effects of stretching and muscle relaxation techniques on proprioceptive receptors, which, together with visual and vestibular inputs, are essential for postural control [89]. These interventions may enhance afferent feedback and sensorimotor integration and, alongside reduced mechanical restrictions in ADF-ROM, improve joint positioning and stability during the anterior reach task. Clinically, these findings suggest that all three interventions may offer short-term benefits for dynamic balance, primarily through improvements in joint mobility and proprioceptive input. However, the relatively small magnitude of these change should be interpreted cautiously, as they may be partially attributable to task familiarization and measurement-related factors rather than solely to the efficacy of the intervention.

### 4.4. Roll-Off Phase of Gait

Statistically significant improvements in rearfoot pressure were observed following the interventions, indicating a possible shift in loading patterns during gait. This redistribution of plantar pressure may reflect a more biomechanically efficient heel strike pattern, one likely facilitated by the improved ankle dorsiflexion observed across all intervention groups. Enhanced dorsiflexion allows the tibia to progress further over the foot during the initial contact and stance, which may contribute to a more natural gait cycle and improving shock absorption [16]. Despite the absence of kinematic, temporal, or energetic assessments, increased rearfoot loading may be interpreted as a shift toward a more functional, symmetrical gait, particularly in individuals previously compensating for limited dorsiflexion via midfoot or forefoot loading [1,90].

However, the changes in midfoot and forefoot pressure, as well as in foot contact duration, were minimal and did not reach statistical significance. This may suggest that the acute effects of soft tissue interventions primarily influence the initial contact phase of gait, with less impact on subsequent phases, such as propulsion. These subtler aspects of gait mechanics might require longer intervention periods or combined training approaches (e.g., gait retraining) to yield meaningful changes. Nevertheless, the observed increase in rearfoot loading supports the hypothesis that even brief myofascial interventions may acutely influence gait mechanics, potentially contributing to more efficient load distribution and reduced compensatory movement patterns. These outcomes could be especially beneficial in clinical populations with limited dorsiflexion, where abnormal plantar pressure profiles are commonly associated with injury risk or overuse pathologies [90].

### 4.5. Limitations

The findings of the present study should be interpreted in light of several limitations. A limitation of the present study is the small sample size (n = 13), which was primarily due to the limited availability of participants presenting with restricted ADF-ROM within the predefined time frame and specific target population. Although post hoc power analysis suggested great achieved power (1 − β > 0.98) for detecting large effects based on the observed effect sizes ($\eta_{p}^{2}$ = 0.885 for ADF-ROM, $\eta_{p}^{2}$ = 0.302 for balance, and $\eta_{p}^{2}$ = 0.482 for rearfoot pressure), this estimate primarily reflects the magnitude of the observed effects rather than the intrinsic robustness of the study design. In small samples, effect sizes may be inflated, and confidence intervals widened, potentially overestimating the true population effect. Therefore, despite the strong statistical power to detect large effects, caution is warranted when interpreting the findings, and replication in larger, more heterogeneous cohorts is necessary to enhance external validity and confirm the stability of the reported outcomes [91].

Moreover, only acute effects were assessed in the present study. Therefore, the durability of the study outcomes over a longer time frame following a single bout of these techniques remains unexplored. Performance-based outcomes may be influenced by motivational or fatigue-related factors, even with the inclusion of standardized rest periods and familiarization trials. In addition, the heterogeneity of the study sample, the lack of assessor blinding, largely inherent to the intervention design, and the absence of a sham or non-intervention control condition represent key methodological limitations, precluding definitive conclusions regarding the absolute efficacy of the interventions beyond temporal or measurement-related effects. Future studies incorporating appropriate control conditions are warranted to strengthen causal inference.

Finally, although a crossover design was employed with a minimum 7-day washout period between interventions to minimize residual effects, the potential for carry-over effects cannot be entirely excluded. However, given that the interventions examined (SSS, FR, and IASTM) are known to induce primarily short-term physiological responses, typically lasting up to ~60 min, and in some cases up to 24 h, particularly for IASTM [24,28,43,44], the likelihood of meaningful carryover effects influencing subsequent conditions is considered negligible.

### 4.6. Clinical Implications

The findings of this study provide important insights for sports performance professionals and healthcare providers (e.g., physical therapists) working with individuals exhibiting mild restrictions in ankle dorsiflexion range of motion (ADF-ROM) [1,90]. The proposed techniques can be safely and effectively applied to enhance ankle joint mobility in individuals with range-of-motion limitations, with IASTM demonstrating particular efficacy, without compromising performance outcomes such as muscular force production or explosive power [38,60]. The observed improvements in dynamic balance may further contribute to enhanced movement control and postural stability when integrated into comprehensive training programs. Additionally, increases in ADF-ROM may positively influence gait mechanics by facilitating more efficient heel strike and tibial progression [40].

These effects may be particularly relevant in athletic contexts where improvements in mobility are desired without compromising performance, as they suggest meaningful implications for lower-limb function, movement efficiency, and potentially injury risk. Although these outcomes were not directly assessed in the present study, they remain practically relevant, enabling practitioners to incorporate these techniques into warm-up or mobility routines without concern for short-term decrements in strength or power. Furthermore, our findings may have potential relevance in rehabilitation contexts where ADF-ROM restrictions due to ankle injury-related muscle stiffness require intervention. However, these findings should be interpreted with caution, as they were derived from a healthy sample and may not be directly generalizable to clinical populations without further investigation.

## 5. Conclusions

This study’s results provide evidence that applying SSS, FR, and IASTM techniques to the posterior compartment of the lower limb can acutely improve ADF-ROM in individuals with limited ankle mobility. Among the interventions examined, IASTM produced the greatest improvement in ADF-ROM, with FR showing comparable effects and SSS demonstrating smaller improvements; however, statistically significant differences were observed only between IASTM and SSS. Minor benefits in A-YBT and RF-PP distribution were observed across all techniques, suggesting a potential role in influencing dynamic balance and gait-related parameters. None of the interventions led to significant improvements in APF-IS or SLCVJ performance.

## Figures and Tables

**Table 1:** Means, and percentage change (confidence intervals in parentheses) of ankle and foot functional outcomes following flexibility and myofascial release interventions applied to the posterior lower leg.

Outcomes	Value	SSS		FR		IASTM	
		Pre	Post	Pre	Post	Pre	Post
ADF-ROM	°	10.8 (9.9, 11.7)	13.2 (11.7, 14.7) *	10.8 (9.9, 11.6)	14.4 (13.6, 15.2) *	10.5 (9.8, 11.2)	15.7 (14.7, 17.0) *†
	%Δ	21.5 (14.4, 28.5)		35.4 (22.9, 48.0)		50.7 (34.4, 66.9)	
APF-IS	N	626.3 (532.7, 719.9)	642.4 (541.6, 743.1)	601.3 (498.9, 703.7)	587.4 (489.6, 685.1)	643.1 (528.9, 757.3)	606.7 (494.1, 719.3)
	%Δ	2.6 (−2.9, 8.1)		−1.3 (−6.3, 3.7)		−5.1 (−11.1, 0.9)	
A-YBT ‡	%LL	79.8 (76.2, 83.5)	80.2 (76.6, 83.9)	78.6 (75.1, 82.1)	80.8 (77.5, 84.2)	80.1 (76.6, 83.7)	81.0 (77.9, 84.1)
	%Δ	0.5 (−0.4, 1.5)		3.0 (−0.5, 6.5)		1.2 (−0.6, 2.9)	
SLCVJ	cm	14.6 (12.0, 17.1)	14.3 (11.6, 17.1)	14.1 (12.1, 16.2)	14.2 (11.8, 16.7)	14.4 (12.3, 16.6)	14.5 (12.0, 16.9)
	%Δ	−1.9 (−7.2, 3.4)		0.2 (−4.3, 4.7)		−0.5 (−4.2, 3.2)	
FC	s	0.71 (0.66, 0.75)	0.69 (0.64, 0.73)	0.71 (0.65, 0.78)	0.70 (0.64, 0.76)	0.70 (0.65, 0.75)	0.70 (0.65, 0.75)
	%Δ	−2.6 (−4.9, −0.2)		−1.3 (−5.8, 3.3)		0.4 (−1.6, 2.3)	
RF-PP ‡	N·cm^−2^	33.7 (29.4, 38.0)	34.6 (29.8, 39.3)	34.4 (28.9, 40.0)	35.5 (30.3, 40.6)	34.2 (29.6, 38.9)	35.2 (29.7, 40.8)
	%Δ	2.2 (−0.8, 5.2)		3.9 (−0.8, 8.5)		2.4 (−0.6, 5.5)	
MD-PP	N·cm^−2^	13.4 (10.6, 16.2)	13.5 (9.3, 17.8)	13.1 (10.4, 15.8)	13.7 (10.8, 16.7)	14.2 (11.4, 17.0)	13.5 (9.8, 17.3)
	%Δ	0.03 (−15.7, 15.7)		7.0 (−4.8, 18.7)		−6.9 (−16.1, 2.2)	
FF-PP	N·cm^−2^	40.8 (35.0, 46.6)	40.2 (34.4, 46.0)	41.6 (36.8, 46.3)	41.5 (36.3, 46.7)	41.6 (36.3 46.8)	39.9 (34.2, 45.5)
	%Δ	−0.5 (−7.6, 6.5)		0.1 (−5.8, 5.9)		−4.4 (−8.4, −0.4)	

Note: SSS = static self-stretching; FR = foam rolling; IASTM = Instrument-Assisted Soft Tissue Mobilization; (%Δ) = percentage change; ADF-ROM = Ankle Dorsiflexion Range of Motion; APF-IS = ankle plantar flexors isometric strength; A-YBT = anterior excursion in the Y-Balance Test; %LL = percentage of leg length; SLCVJ = single-leg countermovement vertical jump; FC = foot contact; RF = rearfoot; MD = midfoot; FF = forefoot; PP = plantar pressure; * significant difference compared to post-intervention assessments (*p* < 0.001). † Significant difference compared to post-SSS (*p* < 0.05); ‡ significant main effects for time (pre–post intervention assessment; *p* < 0.05 for A-YBT and *p* < 0.01 for RF-PP).

## Data Availability

The data presented in this study are available upon request from the corresponding author.

## Abbreviations

The following abbreviations are used in this manuscript:

SSS	Static self-stretching
FR	Foam rolling
IASTM	Instrument-assisted soft tissue mobilization
ADF-ROM	Ankle dorsiflexion range of motion
APF-IS	Ankle plantar flexor isometric strength
SLCVJ	Single-leg countermovement vertical jump
A-YBT	Anterior reach in the Y-Balance Test
%LL	Percentage of leg length
FC	Foot contact
RF-PP	Rearfoot plantar pressure
MD-PP	Midfoot plantar pressure
FF-PP	Forefoot plantar pressure
%Δ	Percentage difference
ANOVA	Analysis of variance
$\eta_{p}^{2}$	Partial eta squared

## References

[1] Almansoof H.S., Nuhmani S., Muaidi Q. (2023). Role of Ankle Dorsiflexion in Sports Performance and Injury Risk: A Narrative Review. Electron. J. Gen. Med..

[2] De Noronha M., Refshauge K.M., Herbert R.D., Kilbreath S.L. (2006). Do Voluntary Strength, Proprioception, Range of Motion, or Postural Sway Predict Occurrence of Lateral Ankle Sprain?. Br. J. Sports Med..

[3] Hoch M.C., Staton G.S., Medina McKeon J.M., Mattacola C.G., McKeon P.O. (2012). Dorsiflexion and Dynamic Postural Control Deficits Are Present in Those with Chronic Ankle Instability. J. Sci. Med. Sport.

[4] Whitting J.W., Steele J.R., McGhee D.E., Munro B.J. (2011). Dorsiflexion Capacity Affects Achilles Tendon Loading during Drop Landings. Med. Sci. Sports Exerc..

[5] Chuckpaiwong B., Cook C., Pietrobon R., Nunley J.A. (2007). Second Metatarsal Stress Fracture in Sport: Comparative Risk Factors between Proximal and Non-Proximal Locations. Br. J. Sports Med..

[6] Nakale N.T., Strydom A., Saragas N.P., Ferrao P.N.F. (2018). Association Between Plantar Fasciitis and Isolated Gastrocnemius Tightness. Foot Ankle Int..

[7] Malliaras P., Cook J.L., Kent P. (2006). Reduced Ankle Dorsiflexion Range May Increase the Risk of Patellar Tendon Injury among Volleyball Players. J. Sci. Med. Sport.

[8] Piva S.R., Goodnite E.A., Childs J.D. (2005). Strength Around the Hip and Flexibility of Soft Tissues in Individuals with and without Patellofemoral Pain Syndrome. J. Orthop. Sports Phys. Ther..

[9] Wahlstedt C., Rasmussen-Barr E. (2015). Anterior Cruciate Ligament Injury and Ankle Dorsiflexion. Knee Surg. Sports Traumatol. Arthrosc..

[10] Backman L.J., Danielson P. (2011). Low Range of Ankle Dorsiflexion Predisposes for Patellar Tendinopathy in Junior Elite Basketball Players: A 1-Year Prospective Study. Am. J. Sports Med..

[11] van Dyk N., Farooq A., Bahr R., Witvrouw E. (2018). Hamstring and Ankle Flexibility Deficits Are Weak Risk Factors for Hamstring Injury in Professional Soccer Players: A Prospective Cohort Study of 438 Players Including 78 Injuries. Am. J. Sports Med..

[12] Abbasi S., Mousavi S.H., Khorramroo F. (2024). Association between Lower Limb Alignment and Low Back Pain: A Systematic Review with Meta-Analysis. PLoS ONE.

[13] Shitara H., Tajika T., Kuboi T., Ichinose T., Sasaki T., Hamano N., Endo T., Kamiyama M., Honda A., Miyamoto R. (2021). Ankle Dorsiflexion Deficit in the Back Leg Is a Risk Factor for Shoulder and Elbow Injuries in Young Baseball Players. Sci. Rep..

[14] Moreno-Pérez V., Delcoso J., Raya-González J., Nakamura F.Y., Castillo D. (2020). Effects of Basketball Match-Play on Ankle Dorsiflexion Range of Motion and Vertical Jump Performance in Semi-Professional Players. J. Sports Med. Phys. Fit..

[15] Moreno-Pérez V., Soler A., Ansa A., López-Samanes Á., Madruga-Parera M., Beato M., Romero-Rodríguez D. (2020). Acute and Chronic Effects of Competition on Ankle Dorsiflexion ROM in Professional Football Players. Eur. J. Sport Sci..

[16] Rao Y., Yang N., Gao T., Zhang S., Shi H., Lu Y., Ren S., Huang H. (2023). Effects of Peak Ankle Dorsiflexion Angle on Lower Extremity Biomechanics and Pelvic Motion during Walking and Jogging. Front. Neurol..

[17] Kunz K.M., Kirk D.G., Wadner J., Martonick N.J.P. (2026). Associations Between Limited Dorsiflexion Under Load and Compensatory Hip/Pelvic Gait Patterns in Healthy Adults. Biomechanics.

[18] Reis e Silva M., Lerebourg L. (2024). The Effect of Ankle Dorsiflexion on Sagittal Posture and Core Muscle Activation. Biomechanics.

[19] Ota S., Ueda M., Aimoto K., Suzuki Y., Sigward S.M. (2014). Acute Influence of Restricted Ankle Dorsiflexion Angle on Knee Joint Mechanics during Gait. Knee.

[20] Guillén-Rogel P., San Emeterio C., Marín P.J. (2017). Associations between Ankle Dorsiflexion Range of Motion and Foot and Ankle Strength in Young Adults. J. Phys. Ther. Sci..

[21] Basnett C.R., Hanish M.J., Wheeler T.J., Miriovsky D.J., Danielson E.L., Barr J.B., Grindstaff T.L. (2013). Ankle Dorsiflexion Range of Motion Influences Dynamic Balance in Individuals with Chronic Ankle Instability. Int. J. Sports Phys. Ther..

[22] Yun S.J., Kim M.-H., Weon J.-H., Kim Y., Jung S.-H., Kwon O.-Y. (2016). Correlation between Toe Flexor Strength and Ankle Dorsiflexion ROM during the Countermovement Jump. J. Phys. Ther. Sci..

[23] Cerrillo-Sanchis J., Ricart-Luna B., Rodrigo-Mallorca D., Muñoz-Gómez E., Domínguez-Navarro F., Mollà-Casanova S., Chulvi-Medrano I. (2024). Relationship between Ankle Dorsiflexion Range of Motion and Sprinting and Jumping Ability in Young Athletes. J. Bodyw. Mov. Ther..

[24] Behm D.G., Blazevich A.J., Kay A.D., McHugh M. (2015). Acute Effects of Muscle Stretching on Physical Performance, Range of Motion, and Injury Incidence in Healthy Active Individuals: A Systematic Review. Appl. Physiol. Nutr. Metab..

[25] Behm D.G., Alizadeh S., Daneshjoo A., Anvar S.H., Graham A., Zahiri A., Goudini R., Edwards C., Culleton R., Scharf C. (2023). Acute Effects of Various Stretching Techniques on Range of Motion: A Systematic Review with Meta-Analysis. Sports Med. Open.

[26] Kalichman L., Ben David C. (2017). Effect of Self-Myofascial Release on Myofascial Pain, Muscle Flexibility, and Strength: A Narrative Review. J. Bodyw. Mov. Ther..

[27] Behm D.G., Wilke J. (2019). Do Self-Myofascial Release Devices Release Myofascia? Rolling Mechanisms: A Narrative Review. Sports Med..

[28] Cheatham S.W., Lee M., Cain M., Baker R., Hills D. (2016). The Efficacy of Instrument Assisted Soft Tissue Mobilization: A Systematic Review. J. Can. Chiropr. Assoc..

[29] Cheatham S.W., Baker R., Kreiswirth E. (2019). Instrument assisted soft-tissue mobilization: A commentary on clinical practice guidelines for rehabilitation professionals. Int. J. Sports Phys. Ther..

[30] Cheatham S.W., Kolber M.J., Cain M., Lee M. (2015). The effects of self-myofascial release using a foam roll or roller massager on joint range of motion, muscle recovery, and performance: A systematic review. Int. J. Sports Phys. Ther..

[31] Chaabene H., Behm D.G., Negra Y., Granacher U. (2019). Acute Effects of Static Stretching on Muscle Strength and Power: An Attempt to Clarify Previous Caveats. Front. Physiol..

[32] Škarabot J., Beardsley C., Štirn I. (2015). Comparing the Effects of Self-Myofascial Release with Static Stretching on Ankle Range-of-Motion in Adolescent Athletes. Int. J. Sports Phys. Ther..

[33] Smith J.C., Washell B.R., Aini M.F., Brown S., Hall M.C. (2019). Effects of Static Stretching and Foam Rolling on Ankle Dorsiflexion Range of Motion. Med. Sci. Sports Exerc..

[34] Aggarwal A., Agarwal N., Rathi M., Palekar T.J. (2024). Effectiveness of Instrument Assisted Soft Tissue Mobilization versus Foam Rolling on Trigger Point Release in Calf Muscles. J. Bodyw. Mov. Ther..

[35] Warneke K., Lohmann L.H. (2024). Revisiting the Stretch-Induced Force Deficit: A Systematic Review with Multilevel Meta-Analysis of Acute Effects. J. Sport Health Sci..

[36] Behm D.G., Chaouachi A. (2011). A Review of the Acute Effects of Static and Dynamic Stretching on Performance. Eur. J. Appl. Physiol..

[37] Jung E.Y., Jung J.H., Cho H.Y., Kim S.H. (2023). Effects of Plantar Flexor Stretching on Static and Dynamic Balance in Healthy Adults. Int. J. Environ. Res. Public Health.

[38] Yana M., Farhoomand B., Güneş M. (2025). Acute Effects of Instrument-Assisted Soft Tissue Mobilization on the Flexibility, Strength, Vertical Jump, and Dynamic Balance Performances of the Plantar Flexor Muscle in Professional Football Players. J. Bodyw. Mov. Ther..

[39] Klaewkasikum K., Patathong T., Angsanuntsukh C., Woratanarat T., Sanguantrakul J., Woratanarat P. (2022). The Ankle Kinematic Reference of Normal Gait Pattern in Thai Adults. Front. Surg..

[40] Aali S., Rezazadeh F., Badicu G., Grosz W.R. (2021). Effect of Heel-First Strike Gait on Knee and Ankle Mechanics. Medicina.

[41] Elias L.J., Bryden M.P., Bulman-Fleming M.B. (1998). Footedness Is a Better Predictor than Is Handedness of Emotional Lateralization. Neuropsychologia.

[42] Kapreli E., Athanasopoulos S., Stavridis I., Billis E., Strimpakos N. (2015). Waterloo Footedness Questionnaire (WFQ-R): Cross-Cultural Adaptation and Psychometric Properties of Greek Version. Physiotherapy.

[43] Nakamura M., Onuma R., Kiyono R., Yasaka K., Sato S., Yahata K., Fukaya T., Konrad A. (2021). The Acute and Prolonged Effects of Different Durations of Foam Rolling on Range of Motion, Muscle Stiffness, and Muscle Strength. J. Sports Sci. Med..

[44] Grieve R., Byrne B., Clements C., Davies L.J., Durrant E., Kitchen O. (2022). The Effects of Foam Rolling on Ankle Dorsiflexion Range of Motion in Healthy Adults: A Systematic Literature Review. J. Bodyw. Mov. Ther..

[45] Tiberio D., Bohannon R.W., Zito M.A. (1989). Effect of Subtalar Joint Position on the Measurement of Maximum Ankle Dorsiflexic. Clin. Biomech..

[46] Krause D.A., Cloud B.A., Forster L.A., Schrank J.A., Hollman J.H. (2011). Measurement of Ankle Dorsiflexion: A Comparison of Active and Passive Techniques in Multiple Positions. J. Sport Rehabil..

[47] Chatziilias V.A., Mandalidis D.G. (2022). Ankle and Foot Function in Female Athletes Involved in In-Water and Dry-Land Sporting Activities. J. Sports Med. Phys. Fit..

[48] Nigro F., Mangia A.L., Vandi A., Di Michele R., Merni F., Fantozzi S. (2017). Sismes IX National Congress. Sport Sci. Health.

[49] Lesinski M., Muehlbauer T., Granacher U. (2016). Concurrent Validity of the Gyko Inertial Sensor System for the Assessment of Vertical Jump Height in Female Sub-Elite Youth Soccer Players. BMC Sports Sci. Med. Rehabil..

[50] Mandalidis D.G., Karagiannakis D.N. (2020). A Comprehensive Method for Assessing Postural Control during Dynamic Balance Testing. MethodsX.

[51] Foldager F.N., Aslerin S., Baekdahl S., Tønning L.U., Mechlenburg I. (2023). Interrater, Test-Retest Reliability of the Y Balance Test. A Reliability Study Including 51 Healthy Participants. Int. J. Exerc. Sci..

[52] Akagi R., Takahashi H. (2013). Acute Effect of Static Stretching on Hardness of the Gastrocnemius Muscle. Med. Sci. Sports Exerc..

[53] Behm D.G., Kibele A. (2007). Effects of Differing Intensities of Static Stretching on Jump Performance. Eur. J. Appl. Physiol..

[54] Wilke J., Müller A.-L., Giesche F., Power G., Ahmedi H., Behm D.G. (2020). Acute Effects of Foam Rolling on Range of Motion in Healthy Adults: A Systematic Review with Multilevel Meta-Analysis. Sports Med..

[55] Hughes G.A., Ramer L.M. (2019). Duration of Myofascial Rolling for Optimal Recovery, Range of Motion, and Performance: A Systematic Review of the Literature. Int. J. Sports Phys. Ther..

[56] Tang S., Sheng L., Wei X., Liang M., Xia J., Chen J. (2025). The Effectiveness of Instrument-Assisted Soft Tissue Mobilization on Pain and Function in Patients with Musculoskeletal Disorders: A Systematic Review and Meta-Analysis. BMC Musculoskelet. Disord..

[57] Cohen J. (2013). Statistical Power Analysis for the Behavioral Sciences.

[58] Morris S.B., DeShon R.P. (2002). Combining Effect Size Estimates in Meta-Analysis with Repeated Measures and Independent-Groups Designs. Psychol. Methods.

[59] Lakens D. (2013). Calculating and Reporting Effect Sizes to Facilitate Cumulative Science: A Practical Primer for t-Tests and ANOVAs. Front. Psychol..

[60] Ikeda N., Otsuka S., Kawanishi Y., Kawakami Y. (2019). Effects of Instrument-Assisted Soft Tissue Mobilization on Musculoskeletal Properties. Med. Sci. Sports Exerc..

[61] Seffrin C.B., Cattano N.M., Reed M.A., Gardiner-Shires A.M. (2019). Instrument-Assisted Soft Tissue Mobilization: A Systematic Review and Effect-Size Analysis. J. Athl. Train..

[62] Morse C.I., Degens H., Seynnes O.R., Maganaris C.N., Jones D.A. (2008). The Acute Effect of Stretching on the Passive Stiffness of the Human Gastrocnemius Muscle Tendon Unit. J. Physiol..

[63] Yoshimura A., Inami T., Schleip R., Mineta S., Shudo K., Hirose N. (2021). Effects of Self-Myofascial Release Using a Foam Roller on Range of Motion and Morphological Changes in Muscle: A Crossover Study. J. Strength Cond. Res..

[64] Shah R., Samuel M.W., Son J. (2023). Acute and Chronic Effects of Static Stretching on Neuromuscular Properties: A Meta-Analytical Review. Appl. Sci..

[65] Schleip R., Duerselen L., Vleeming A., Naylor I.L., Lehmann-Horn F., Zorn A., Jaeger H., Klingler W. (2012). Strain Hardening of Fascia: Static Stretching of Dense Fibrous Connective Tissues Can Induce a Temporary Stiffness Increase Accompanied by Enhanced Matrix Hydration. J. Bodyw. Mov. Ther..

[66] Budini F., Christova M., Gallasch E., Rafolt D., Tilp M. (2018). Soleus H-Reflex Inhibition Decreases during 30 s Static Stretching of Plantar Flexors, Showing Two Recovery Steps. Front. Physiol..

[67] Skinner B., Moss R., Hammond L. (2020). A Systematic Review and Meta-Analysis of the Effects of Foam Rolling on Range of Motion, Recovery and Markers of Athletic Performance. J. Bodyw. Mov. Ther..

[68] Blazevich A.J., Kay A.D., Waugh C., Fath F., Miller S., Cannavan D. (2012). Plantarflexor Stretch Training Increases Reciprocal Inhibition Measured during Voluntary Dorsiflexion. J. Neurophysiol..

[69] Lambert M., Hitchcock R., Lavallee K., Hayford E., Morazzini R., Wallace A., Conroy D., Cleland J. (2017). The Effects of Instrument-Assisted Soft Tissue Mobilization Compared to Other Interventions on Pain and Function: A Systematic Review. Phys. Ther. Rev..

[70] Zuil-Escobar J.C., Martínez-Cepa C.B., Martín-Urrialde J.A., Gómez-Conesa A. (2016). The Prevalence of Latent Trigger Points in Lower Limb Muscles in Asymptomatic Subjects. PM&R.

[71] Grieve R., Clark J., Pearson E., Bullock S., Boyer C., Jarrett A. (2011). The Immediate Effect of Soleus Trigger Point Pressure Release on Restricted Ankle Joint Dorsiflexion: A Pilot Randomised Controlled Trial. J. Bodyw. Mov. Ther..

[72] Kay A.D., Blazevich A.J. (2012). Effect of Acute Static Stretch on Maximal Muscle Performance. Med. Sci. Sports Exerc..

[73] McHugh M.P., Cosgrave C.H. (2010). To Stretch or Not to Stretch: The Role of Stretching in Injury Prevention and Performance. Scand. J. Med. Sci. Sports.

[74] Wiewelhove T., Döweling A., Schneider C., Hottenrott L., Meyer T., Kellmann M., Pfeiffer M., Ferrauti A. (2019). A Meta-Analysis of the Effects of Foam Rolling on Performance and Recovery. Front. Physiol..

[75] Rubini E.C., Costa A.L.L., Gomes P.S.C. (2007). The Effects of Stretching on Strength Performance. Sports Med..

[76] MacDonald N., Baker R., Cheatham S.W. (2016). The effects of instrument assisted soft tissue mobilization on lower extremity muscle performance: A randomized controlled trial. Int. J. Sports Phys. Ther..

[77] Stroiney D.A., Mokris R.L., Hanna G.R., Ranney J.D. (2020). Examination of Self-Myofascial Release vs. Instrument-Assisted Soft-Tissue Mobilization Techniques on Vertical and Horizontal Power in Recreational Athletes. J. Strength Cond. Res..

[78] Gregory J.E., Morgan D.L., Allen T.J., Proske U. (2007). The Shift in Muscle’s Length-Tension Relation after Exercise Attributed to Increased Series Compliance. Eur. J. Appl. Physiol..

[79] Toninelli N., Coratella G., Longo S., Romani G.M., Doria C., Rampichini S., Limonta E., Esposito F., Cè E. (2024). Synergistic Difference in the Effect of Stretching on Electromechanical Delay Components. PLoS ONE.

[80] Mylle I., Funaro A., Crouzier M., Bogaerts S., Vanwanseele B. (2024). Achilles Tendon Compliance Influences Tendon Loading More than Achilles Tendon Twist in Achilles Tendinopathy: A Musculoskeletal Modeling Approach. Front. Bioeng. Biotechnol..

[81] Longo S., Cè E., Bisconti A.V., Rampichini S., Doria C., Borrelli M., Limonta E., Coratella G., Esposito F. (2021). The Effects of 12 Weeks of Static Stretch Training on the Functional, Mechanical, and Architectural Characteristics of the Triceps Surae Muscle–Tendon Complex. Eur. J. Appl. Physiol..

[82] Budini F., Christova M., Gallasch E., Kressnik P., Rafolt D., Tilp M. (2018). Transient Increase in Cortical Excitability Following Static Stretching of Plantar Flexor Muscles. Front. Physiol..

[83] Borisavljević A., Ćosić M., Janković G., Radić I., Janković D., Dopsaj M. (2025). Vibration Foam Rolling Treatment Influence on Acute Changes in Plantar Flexors Muscle Temperature and Surface Emg Activity in Amateur Male Athletes. J. Funct. Morphol. Kinesiol..

[84] Halperin I., Aboodarda S.J., Button D.C., Andersen L.L., Behm D.G. (2014). Roller Massager Improves Range of Motion of Plantar Flexor Muscles without Subsequent Decreases in Force Parameters. Int. J. Sports Phys. Ther..

[85] Rafagnin C.Z., Ferreira A.d.S., Telles G.F., de Carvalho T.L., Alexandre D.J.d.A., Nogueira L.A.C. (2023). Anterior Component of Y-Balance Test Is Correlated to Ankle Dorsiflexion Range of Motion in Futsal Players: A Cross-Sectional Study. Physiother. Res. Int..

[86] Olszewski M., Zając B., Mika A., Golec J. (2024). Ankle Dorsiflexion Range of Motion and Hip Abductor Strength Can Predict Lower Quarter Y-Balance Test Performance in Healthy Males. J. Bodyw. Mov. Ther..

[87] Lima B.N., Lucareli P.R.G., Gomes W.A., Silva J.J., Bley A.S., Hartigan E.H., Marchetti P.H. (2014). The Acute Effects of Unilateral Ankle Plantar Flexors Static- Stretching on Postural Sway and Gastrocnemius Muscle Activity during Single-Leg Balance Tasks. J. Sports Sci. Med..

[88] Coratella G., Longo S., Rampichini S., Doria C., Borrelli M., Limonta E., Michielon G., Cè E., Esposito F. (2021). Passive Stretching Decreases Muscle Efficiency in Balance Tasks. PLoS ONE.

[89] Horak F.B. (2006). Postural Orientation and Equilibrium: What Do We Need to Know about Neural Control of Balance to Prevent Falls?. Age Ageing.

[90] Michalina B., Ida W., Katarzyna K., Brzuszkiewicz-Kuźmicka G., Andrzej W. (2017). Mechanisms of Compensation in the Gait of Patients with Drop Foot. Clin. Biomech..

[91] Button K.S., Ioannidis J.P.A., Mokrysz C., Nosek B.A., Flint J., Robinson E.S.J., Munafò M.R. (2013). Power Failure: Why Small Sample Size Undermines the Reliability of Neuroscience. Nat. Rev. Neurosci..

